# Verification and Analysis of Sheep Tail Type-Associated *PDGF-D* Gene Polymorphisms

**DOI:** 10.3390/ani10010089

**Published:** 2020-01-06

**Authors:** Qing Li, Zengkui Lu, Meilin Jin, Xiaojuan Fei, Kai Quan, Yongbin Liu, Lin Ma, Mingxing Chu, Huihua Wang, Caihong Wei

**Affiliations:** 1 Key Laboratory of Animal Genetics and Breeding and Reproduction of Ministry of Agriculture, Institute of Animal Science, Chinese Academy of Agricultural Sciences, Beijing 100193, China; liqing_0507@163.com (Q.L.); jmlingg@163.com (M.J.); 18409481571@163.com (X.F.); 18633082661@163.com (L.M.); mxchu@263.net (M.C.); 2 Lanzhou Institute of Husbandry and Pharmaceutical Sciences, Chinese Academy of Agricultural Sciences, Lanzhou 730050, China; luzengkui@caas.cn; 3 College of Animal Science and Technology, Henan University of Animal Husbandry and Economy, Zhengzhou 450046, China; quankai1115@163.com; 4 Inner Mongolia Academy of Animal Husbandry Science, Hohhot 010031, China

**Keywords:** *PDGF-D*, sheep, tail type, SNPs, preadipocytes

## Abstract

**Simple Summary:**

*PDGF-D* can be considered a candidate gene for selection for sheep tail type. This study investigated genetic variation of the *PDGF-D* gene in sheep with different tail types verified at a cellular level and revealed the molecular mechanism of *PDGF-D* in sheep tail fat deposition. We detected a total of two SNPs among 533 sheep. g.4122606 C > G site was significantly correlated with tail length, and g.3852134 C > T site was significantly correlated with tail width. In addition, overexpression of *PDGF-D* in sheep preadipocytes can promote adipogenic differentiation. The *PDGF-D* gene may participate in sheep tail fat deposition and could be used for molecular marker-assisted selection of sheep tail type.

**Abstract:**

The aim of this study was to examine the correlation between the platelet-derived growth factor-D (*PDGF-D*) gene and sheep tail type character and explore the potential underlying mechanism. A total of 533 sheep were included in this study. Polymorphic sites were examined by Pool-seq, and individual genotype identification and correlation analysis between tail type data were conducted using the matrix-assisted laser desorption/ionization time-of-flight mass spectrometer (MALDI-TOF-MS) method. JASPART website was used to predict transcription factor binding sites in the promoter region with and without *PDGF-D* gene mutation. The effect of *PDGF-D* on adipogenic differentiation of sheep preadipocytes was investigated. Two single nucleotide polymorphism sites were identified: g.4122606 C > G site was significantly correlated with tail length, and g.3852134 C > T site was significantly correlated with tail width. g.3852134 C > T was located in the promoter region. Six transcription factor binding sites were eliminated after promoter mutation, and three new transcription factor binding sites appeared. Expression levels of peroxisome proliferator-activated receptor gamma (*PPARγ*) and lipoproteinlipase (*LPL*) were significantly up-regulated upon *PDGF-D* overexpression. Oil red O staining showed increased small and large oil drops in the *PDGF-D* overexpression group. Together these results indicate the *PDGF-D* gene is an important gene controlling sheep tail shape and regulating sheep tail fat deposition to a certain degree.

## 1. Introduction

Current studies have indicated that fat-tailed sheep developed from thin-tailed sheep [1]. The fat tail was believed to serve as an important energy reserve to adapt to harsh conditions, such as dry seasons, extreme cold winters, and food shortage [2,3,4,5]. After long-term artificial and natural selective breeding for different morphologies of ovine tails, sheep tails can be classified into five types: short-fat tail, long-fat tail, short-thin tail, long-thin tail, and fat-rumped breeds [6]. However, with the improvement of human living standards, the feeding conditions improved and dietary habits changed, thereby reducing the dependency on the fat tail as an energy reserve [3]. Furthermore, fat-tailed sheep started to show low rates of reproduction and increased feed cost. Identifying the key genes that control lipid metabolism in sheep tail could not only increase economic benefit in livestock but also provide useful information for research into fat deposition and energy storage in obesity.

Platelet-derived growth factor-D (*PDGF-D*), a novel member of the PDGF family, regulates many biological processes, including angiogenesis, tissue fibrosis, tumorigenesis, and lipid metabolism [7,8,9,10]. In previous studies, we examined the selection signals of different tail type sheep and found that *PDGF-D* is strongly selected, indicating *PDGF-D* may be involved in fat deposition in sheep tail or related to sheep tail type [11,12,13]. Other scientists have similarly found that *PDGF-D* has the strongest selection signal in different tailed sheep [14,15,16]. However, no studies have been conducted to reveal the molecular mechanism of *PDGF-D* in the formation of sheep tails.

In this study, we examined a group of sheep comprising 208 Hu sheep, 171 Tibetan sheep, and 154 hybrid sheep (Dorper × Hu) as the research objects. Flight mass spectrometry genotyping technology was used to detect the polymorphic site of the *PDGF-D* gene and associated analysis with sheep tail length, tail width, and tail circumference. Changes of transcription factor binding sites in the promoter region of PDGF-D gene with and without mutations were analyzed. The function of PDGF-D gene in fat deposition of the sheep tail was also examined. This study will improve our understanding about PDGF-D gene contribution in the marker-assisted selection for tail type characteristics of sheep.

## 2. Materials and Methods

### 2.1. Animals and Sample Collection

All the experimental procedures mentioned in the present study were approved by the Science Research Department (in charge of animal welfare issue) of the Institute of Animal Sciences, Chinese Academy of Agricultural Sciences (IAS-CAAS) (Beijing, China). Ethical approval on animal survival was given by the animal ethics committee of IAS-CAAS (No. IASCAAS-AE-03, 12 December 2016). A total of 533 sheep (24 months old), consisting of 208 Hu sheep (short-fat-tailed sheep, Wuwei, Gansu, China), 171 Tibetan sheep (short-thin-tailed sheep, Tianzhu, Gansu, China), and 154 Dorper-Hu hybrid sheep (intermediate type, Luoyang, Henan, China) were used as the experimental population for the association analysis. Animals from the same breed originated from the same farm. Blood samples were collected from all sheep. The length, width, and circumference of tail from each sheep were measured and recorded.

### 2.2. PCR Amplification and Mass Array Genotyping

DNA was extracted from whole blood samples using the TIANamp Genomic DNA Kit (TIANGEN Biotech, Beijing, China) according to the manufacturer’s instructions. The quantity and quality of the extracted DNA were measured using a NanoDrop 2000 spectrophotometer (Thermo, Waltham, MA, USA) and by gel electrophoresis, respectively. We constructed two DNA pools (50 ng/μL/sheep) to identify potential single nucleotide polymorphisms (SNPs). One pool was composed of 30 Tibetan sheep samples that were selected randomly. The other consisted of 30 Hu sheep samples that were selected randomly. Fifteen pairs of primers were designed using Primer 3.0 to amplify all exons of the PDGF-D gene and 1000 bp of flanking sequences based on the reference sequence. The primer pairs are listed in Table S1.

The PCR reaction mixture (25 μL) consisted of 12.5 µL 2× Taq PCR Master Mix, 1 µL Primer-F, 1 µL Primer-R, 2 μL pooled DNA, and 8.5 μL ddH_2_O. The PCR reaction was as follows: 94 °C for 5 min; 95 °C 30 s, T_m_ 30 s, 72 °C 30 s, for 35 cycles; and 72 °C for 10 min. PCR products were examined on a 2% agarose gel. PCR products were then sequenced by BoMiao Biological Technology Co., Ltd. (Beijing, China), and the sequences were compared using DNAMAN 6.0 software (https://www.lynnon.com) and Chromas 2 software (http://technelysium.com.au/wp/chromas) to detect potential SNPs.

The identified SNPs were genotyped in the experimental population using matrix-assisted laser desorption/ionization time-of-flight mass spectrometer (MALDI-TOF-MS, Thermo, Waltham, MA, USA). First, based on SNP locus information, single-base amplification and extension primers of the site to be tested were designed using Sequenom’s Assay Design 3.1 (iPlex assay, Sequenom, San Diego, CA, USA). Subsequently, the remaining dNTPs in PCR products were removed using shrimp alkaline phosphatase enzyme (SAP, Agena, San Diego, CA, USA). Third, a single base extension reaction was performed. Finally, after the sample was purified by clean resin (Sequenom), the purified product was spotted using a Mass ARRAY Nano dispenser (Sequenom), transferred to a Spectro CHIP (Sequenom), and analyzed by MALDI-TOF-MS (Sequenom).

### 2.3. Population Genetic Analysis of Polymorphisms in the PDGF-D Gene

Microsoft Excel 2013 (Microsoft Inc., Redmond, WA, USA) was used to calculate the allele frequencies, polymorphic information content (PIC), heterozygosity (He), and effective number of allele (Ne). The Hardy–Weinberg equilibrium was tested for each site through the chi-square test. Association analysis between SNP genotypes and/or haplotypes and three tail traits were conducted by SAS 9.2 software, based on the following animal model: y_i_ = μ + G_i_ + b_i_ + e_i_, in which y_i_ was the trait measured in individual, μ was the overall mean, G_i_ was a fixed effect corresponding to the genotype of polymorphisms, b_i_ was the breed effect, and e_i_ was a random residual effect.

### 2.4. Bioinformatics Analysis

The promoter and coding sequences of the PDGF-D gene were obtained from NCBI (https://www.ncbi.nlm.nih.gov/). The structures of transcriptional factor (TF) binding sites before and after mutations in promoter regions were also evaluated using JASPAR (http://jaspar.binf.ku.dk/cgi-bin/jaspar_db.pl).

### 2.5. Cell Culture and Transfection

Sheep preadipocytes were isolated from the tail fat of a 70-day-old fetus in Hu sheep as described by Cai et al. [17]. The cells were seeded in 6-well plates overnight and cultured in DEME/F12 (Gibco, Grand Island, NY, USA) supplemented with 10% fetal bovine serum (FBS, Hyclone, Logan, UT, USA) and 2% penicillin/streptomycin at 37 °C in a humidified 5% CO_2_ incubator. The following day, cells were cultured in a new fresh medium containing the PDGF-D-overexpression lentiviral vector (Genechem, Shanghai, China) for 12 h. Preadipocytes were then cultured in new differentiation medium containing 10% FBS, 1 μM dexamethasone (Macklin, Shanghai, China), 0.5 mM isobutylmethylxanthine (Macklin), and 10 mg/mL insulin (Macklin) for 2 days, followed by 10 mg/mL insulin alone for 2 days. Virus without PDGF-D overexpression served as negative control. The date that cells were cultured with differentiation medium was set as the first day (1 d).

### 2.6. Quantitative Real-Time PCR Analysis

Cells were collected from the PDGF-D group and negative control group at various times (0, 1, 3, 5, and 7 d). Total RNA was extracted from cells using the TRIzol (Invitrogen, Carlsbad, CA, USA) method, and cDNA was synthesized from total RNA. qRT-PCR was performed using the TransStart Green qPCR SuperMix (TransStart Green, Beijing, China) and 480 II LightCycler instrument (Roche, Basel, Switzerland), and samples were analyzed in triplicate. The 2^−ΔΔ*C*t^ method was used to calculate the relative expression of target genes, and ACTB (beta-actin) served as the reference gene. qRT-PCR primer information is listed in Table S2.

### 2.7. Oil Red O Staining

Oil red O dye was produced by combining saturated oil red O original solution (Biotopped, Beijing, China) and distilled water in a ratio of 3:2 and filtering the mixture. Cells differentiated for 7 days were washed twice with PBS, fixed with 4% paraformaldehyde for 20 min, and then rinsed with distilled water. Oil red O dye was added, and cells were incubated for 10 min. Stained cells were then rinsed with distilled water 2–3 times and observed and photographed under a microscope.

### 2.8. Statistical Analysis

All data reported are expressed as mean ± SE. Student’s *t*-test was carried out using SPSS software (SPSS 17.0, Chicago, IL, USA) for statistical analysis of the data. A *p* value of < 0.05 was considered to be statistically significant.

## 3. Results

### 3.1. SNP Detection and Genotyping

**Figure 1:**
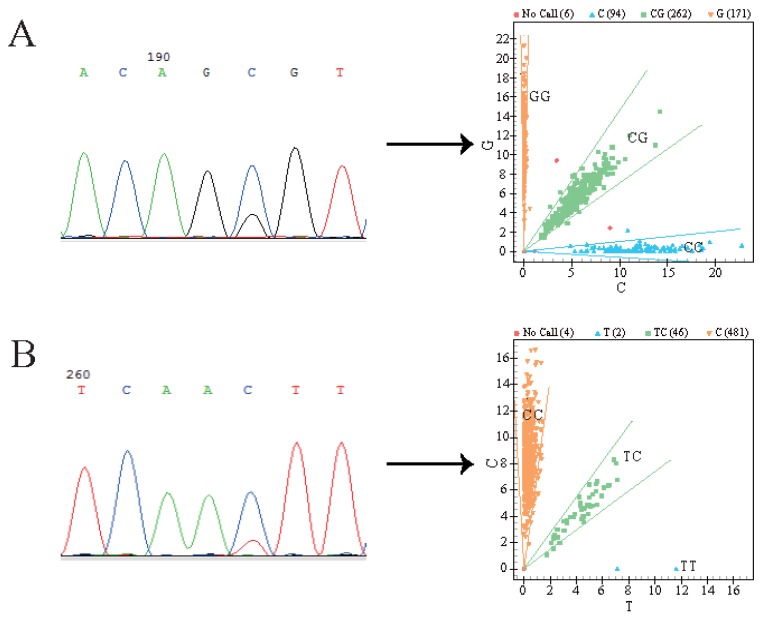
P*DGF-D* gene pool sequencing and genotyping results. (**A**) Site of g.4122606 C > G. (**B**) Site of g.3852134 C > T. Yellow region, blue region, and green region represent different genotypes. Numbers in brackets indicate number of individuals of the three genotypes.

The sequencing results revealed two SNPs in the PDGF-D gene (Figure 1): g.4122606 C > G and g.3852134 C > T. Both SNPs were genotyped and classified into three genotypes in the experimental population consisting of 208 Hu sheep, 171 Tibetan sheep, and 154 Dorper-Hu hybrid sheep (Figure 1).

### 3.2. Genetic Parameters Calculation

The genetic parameters calculation results are shown in Table 1. All sites were in Hardy–Weinberg status (*p* > 0.05). g.4122606 C > G was in moderate *PIC* status (0.25 < PIC < 0.5). The *He* and *Ne* of g.4122606 C > G were 0.47–0.5 and 1.88–2.0, respectively. The g.4122606 C > G site was highly variable. In addition, the alleles of g.4122606 C > G were evenly distributed. However, g.3852134 C > T showed low *PIC* status (PIC < 0.25), and the He and Ne were lower than those of g.4122606 C > G. These data showed that g.3852134 C > T was in a less variable status, and the alleles were unevenly distributed.

### 3.3. Correlation Analysis between PDGF-D Gene Polymorphism and Tail Type in Sheep

The association analysis results revealed that g.4122606 C > G was significantly related with tail length (*p* < 0.05, Table 2). The tail length of sheep with the GG genotype was significantly shorter than CC and CG genotype carriers. No differences were observed with tail width and circumference. However, g.3852134 C > T was significantly related with the tail width (*p* < 0.05, Table 2). Regarding g.4122606 C > G, the CC genotype carriers had the smallest tail length, width, and circumference. Particularly, the tail width of sheep with CC genotype was significantly narrower than TT genotypes (*p* < 0.05). However, the tail length and circumference showed no differences between genotypes.

### 3.4. Transcriptional Factor Binding Sites Prediction

The mutation g.3852134 C > T occurred in the promoter region of PDGF-D. The promoter sequence obtained from NCBI was used for predicting TF binding sites. Multiple TF binding sites were present in the promoter region, such as those for insulinoma-associated protein 1 (INSM1), homebox (HOXA5), and double homeobox 4 (DUX4) (Table 3). In addition, several TF binding sites were absent after mutation, such as those for hepatocyte nuclear factor 4γ (HNF4G) and human intestinal trefoil factor (HItf) (Table 3). Some new TF binding sites also appeared as a result of mutation, such as those for CCAAT/enhancer binding protein A (CEBPA) and Jun (Table 3).

### 3.5. Lentiviral Overexpression Efficiency Assay

We next used a lentivirus overexpressing PDGF-D. At 72 h after transfection, approximately 70% of cells expressed the GFP reporter gene (Figure S1A,B), indicating that PDGF-D had been successfully integrated into chromosomes. qRT-PCR results showed that the PDGF-D gene was expressed at significantly higher levels in infected cells than levels in the negative controls at every time point (Figure S1C).

### 3.6. Influence of Overexpressed PDGF-D on the Expression of Adipose Differentiation-Related Marker Genes

**Figure 2:**
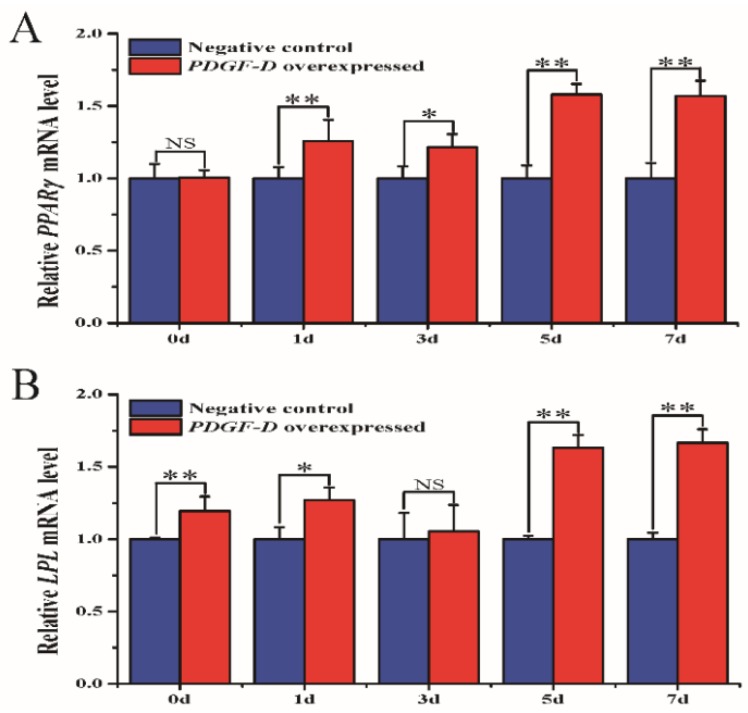
mRNA expression of the *PPARγ* and *LPL* genes. (**A**) mRNA expression of the *PPARγ* gene. (**B**) mRNA expression of the *LPL* gene. * *p* < 0.05, ** *p* < 0.01, NS: no significant difference.

We next detected the expression level of *PPARγ* (peroxisome proliferator-activated receptor gamma) and *LPL* (lipoproteinlipase) genes, which are marker genes in adipogenesis (Figure 2). Overexpression of *PDGF-D* resulted in up-regulated expression level of these marker genes. The relative mRNA expression level of *PPARγ* was significantly higher than that of the negative control at 1, 5, and 7 d (*p* < 0.01), as well as at 3 d (*p* < 0.05). The relative mRNA expression level of *LPL* was significantly higher than that of the negative control at 0, 5, and 7 d (*p* < 0.01), as well as 1 d (*p* < 0.05). No significant difference was observed in the expression level of *LPL* at 3 d.

### 3.7. Oil Red O Staining

**Figure 3:**
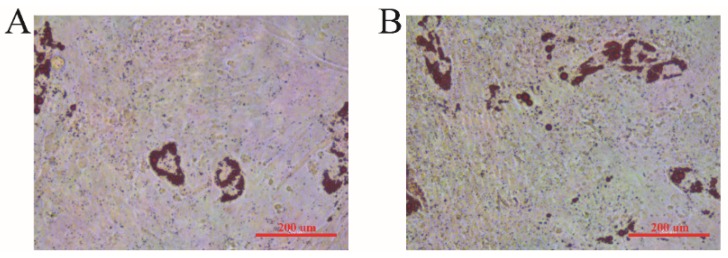
Oil red O staining when overexpressing the *PDGF-D* gene in sheep preadipocytes after 7 days of induced differentiation. (**A**) Control group. (**B**) *PDGF-D* overexpressed group.

We performed oil red O staining on sheep preadipocytes on day 7 after induction of differentiation. As shown in Figure 3, many little lipid drops were stained in red, and the lipid ring was observed. The number of lipid drops in the PDGF-D overexpression group was higher than that in the negative control. These results suggested that PDGF-D could promote the formation of lipid drops in adipocytes in vitro.

## 4. Discussion

Many studies have suggested that *PDGF-D* may be associated with tail type [11,12,13,14,15,16]. However, few studies have conducted functional verification of *PDGF-D* in controlling the formation of sheep tails. In our research, we identified two SNPs in the *PDGF-D* gene that were related to tail traits. The g.4122606 C > G mutation was associated with tail length, and this mutation occurs in an intron region. While introns are not expressed as protein, they play a crucial role in transcriptional regulation [18], such as by encoding microRNA to regulate target genes [19], performing as promoters or enhancers, and participating in alternative splicing [20,21,22,23,24]. The g.4122606 C > G locus showed moderate polymorphism, relatively high heterozygosity, and a large degree of genetic variation, which could bring more selection effects. The g.3852134 C > T locus has lower polymorphism, lower heterozygosity, and less genetic variation, which may be related to the highly selective breeding of the experimental population during breeding. Compared with the g.4122606 C > G locus, the number of effective alleles at the g.3852134 C > T locus is smaller, indicating the uneven distribution of the g.3852134 C > T locus in the test population may be related to the selection and matching system of the test population.

TFs play significant roles in regulating gene expression. The mutation g.3852134 C > T located in the promoter region was associated with tail width. Previous studies showed that mutations in promoter regions may change TF binding sites [25,26,27]. Our results showed that some new TF binding sites appeared in the promoter harboring mutations, such as sites for C/EBPα and Jun. C/EBPα functions to trigger differentiation of preadipocytes into mature adipocytes [28,29]. Some studies showed the slight increase in C/EBPα expression after the decrease of C/EBPβ and C/EBPδ before the expression of adipocyte-specific genes [30,31]. C/EBP families participate in the early stage in adipogenesis. As a pleiotropic transcriptional activator, C/EBPα transactivates promoters from numerous adipocyte genes [4]. In addition, some reports showed that forced expression of C/EBPα in 3T3-L1 preadipocytes stimulated adipogenesis without any hormonal induction [2,3,5]. In contrast, blocking expression of C/EBPα inhibited adipogenesis [32]. Furthermore, some studies showed that C/EBPα is a key regulator related with insulin sensitivity. These observations demonstrated that C/EBPα is required for preadipocyte differentiation. Jun and Fos form a dipolymer named AP-1 (activator protein-1), which is an important positive regulator in adipogenesis.

Previous studies indicated that the PDGF family can promote preadipocyte proliferation and inhibit preadipocyte differentiation [33,34]. Another study showed that *PDGF-BB* can promote the adipogenic differentiation of fibroblasts [35]. These results indicate that the PDGF family participates in fat metabolism. In addition, the expression levels of adipose differentiation marker genes (*PPARγ* and *LPL*) significantly increased after the overexpression of *PDGF-D* in sheep preadipocytes, indicating *PDGF-D* plays a certain role in the tail fat deposition process of the sheep. Oil red O revealed increased accumulation of big and small lipid droplets after the overexpression of *PDGF-D* compared with the control group, which further proved the important role of *PDGF-D* in the tail fat deposition process of t sheep.

*PDGF-D* promotes mitosis, proliferation and division of vascular smooth muscle cells, and formation of new blood vessels [36]. The sheep tail is rich in blood capillaries. Angiogenesis and organofaction are two closely related processes in that the former is the premise of the latter. Oxygen and nutriments can be delivered to tissue after angiopoiesis. Whether *PDGF-D* participates in angiopoiesis and then adipogenesis or if it directly controls fat development should be examined in further studies. In addition, studies have shown that *PDGF-D* is related to sheep body size in various environments, and *PDGF-D* may also directly regulate sheep tail size [37].

## 5. Conclusions

The *PDGF-D* gene shows polymorphisms in sheep. g.4122606 C > G was significantly correlated with tail length and g.3852134 C > T was significantly correlated with tail width. In addition, overexpressed *PDGF-D* in sheep preadipocytes can promote adipogenic differentiation. This result can be applied to molecular marker-assisted selection of sheep tail type.

## Figures and Tables

**Table 1:** Genetic parameter calculation of g.4122606 C > G and g.3852134 C > T.

Locus	Breed	Genotype	Genotype Frequency	Allele Frequency	*He*	*Ne*	*PIC*	Hardy–Weinberg Test (*p*-Value)
g.4122606 C > G	Hu sheep	CC (23)	0.110577	C (0.38) G (0.63)	0.47	1.88	0.36	0.064457718
		CG (110)	0.528846					
		GG (75)	0.360577					
	Tibetan sheep	CC (49)	0.286550	C (0.52) G (0.48)	0.5	2.0	0.37	0.50856548
		CG (81)	0.473684					
		GG (41)	0.239766					
	Hybrid sheep	CC (24)	0.155844	C (0.39) G (0.61)	0.48	1.91	0.36	0.832708982
		CG (72)	0.467532					
		GG (58)	0.376623					
g.3852134 C > T	Hu sheep	CC (187)	0.899038	C (0.94) T (0.06)	0.10	1.12	0.10	0.070148151
		CT (19)	0.091346					
		TT (2)	0.009615					
	Tibetan sheep	CC (157)	0.918129	C (0.96) T (0.04)	0.08	1.09	0.08	0.576740804
		CT (14)	0.081871					
		TT (0)	0.000000					
	Hybrid sheep	CC (141)	0.915584	C (0.96) T (0.04)	0.08	1.09	0.08	0.584470126
		CT (13)	0.084416					
		TT (0)	0.000000					

**Table 2:** Association analysis of *PDGF-D* gene with tail traits.

Locus	Genotype	Tail Length (cm)	Tail Width (cm)	Tail Circumference (cm)
g.4122606 C > G	CC	19.940 ± 0.276 ^a^	9.897 ± 0.202	20.424 ± 0.414
	CG	19.733 ± 0.166 ^a,b^	9.751 ± 0.121	20.230 ± 0.249
	GG	19.168 ± 0.204 ^b^	9.480 ± 0.149	19.611 ± 0.306
g.3852134 C > T	CC	19.543 ± 0.123	9.657 ± 0.089 ^a^	20.026 ± 0.183
	CT	20.060 ± 0.396	9.912 ± 0.288 ^a,b^	20.257 ± 0.590
	TT	19.581 ± 1.903	12.996 ± 1.382 ^b^	25.756 ± 2.837

Note: The size of the sheep’s tail in adulthood (tail length, tail width, and tail circumference) is basically determined, and the difference within the same variety is small. Therefore, we finally merged the three breeds of sheep into one large group for correlation analysis. In the same column, values with different lower-case letters are significantly different (*p* < 0.05).

**Table 3:** Transcriptional factor binding prediction in the *PDGF-D* promoter region with mutation at g.3852134 C > T.

Group	Model ID	Melel Name	Score	Relative Score	Start	End	Strand	Predicted Site Sequence
Transcriptional factor binding sites before mutation	MA0484.1	HNF4G	5.173	0.81593069560891	884	898	−1	GAAGTTGAGGGGGCA
	MA0155.1	INSM1	9.035	0.833563663883174	884	895	−1	GTTGAGGGGGCA
	MA0504.1	NR2C2	10.447	0.852923410523265	884	898	−1	GAAGTTGAGGGGGCA
	MA0528.1	ZNF263	7.665	0.818705635466418	884	904	−1	GGTGAAGAAGTTGAGGGGGCA
	MA0503.1	Nkx2-5	4.672	0.826755938995108	885	895	−1	GCCCCCTCAAC
	MA0528.1	ZNF263	8.684	0.827686489190624	885	905	−1	AGGTGAAGAAGTTGAGGGGGC
	MA0027.1	En1	4.660	0.810707218857438	887	897	−1	AAGTTGAGGGG
	MA0158.1	HOXA5	4.749	0.82019043611391	890	897	−1	CTCAACTT
	MA0130.1	ZNF354C	4.636	0.812679270758179	890	895	−1	CTCAAC
	MA0468.1	DUX4	0.491	0.811042995629078	892	902	−1	CAACTTCTTCA
	MA0109.1	Hltf	5.162	0.86314820468518	892	901	−1	CAACTTCTTC
	MA0080.3	Spi1	8.605	0.859723524619004	892	906	−1	AAGGTGAAGAAGTTG
	MA0466.1	CEBPB	−1.589	0.803283232723208	893	903	1	AACTTCTTCAC
	MA0158.1	HOXA5	4.377	0.807088931935232	893	900	−1	AAGAAGTT
	MA0598.1	EHF	6.510	0.869437914477032	894	901	1	ACTTCTTC
Transcriptional factor binding sites after mutation	MA0155.1	INSM1	7.803	0.807735310463782	884	895	−1	ATTGAGGGGGCA
	MA0528.1	ZNF263	7.211	0.814704352256615	884	904	−1	GGTGAAGAAATTGAGGGGGCA
	MA0528.1	ZNF263	7.080	0.813549796969205	885	905	−1	AGGTGAAGAAATTGAGGGGGC
	MA0158.1	HOXA5	4.749	0.82019043611391	890	897	1	CTCAATTT
	MA0063.1	Nkx2-5	6.260	0.876914864771809	891	897	1	TCAATTT
	MA0468.1	DUX4	1.215	0.818924066022953	892	902	1	CAATTTCTTCA
	MA0075.1	Prrx2	4.766	0.819222411057098	892	896	−1	AATTG
	MA0080.3	Spi1	4.762	0.817634453369339	892	906	−1	AAGGTGAAGAAATTG
	MA0466.1	CEBPB	8.714	0.911280728265531	893	903	1	AATTTCTTCAC
	MA0158.1	HOXA5	6.700	0.888902894857458	893	900	−1	AAGAAATT
	MA0102.3	CEBPA	8.325	0.897723669989696	894	904	1	ATTTCTTCACC
	MA0488.1	JUN	3.746	0.828518034950643	894	906	−1	AAGGTGAAGAAAT

## Supplementary Materials

The following are available online at https://www.mdpi.com/2076-2615/10/1/89/s1, Table S1: Primers used in this study for PCR, Table S2: Primers used in this study for qRT-PCR, Table S3: The breed effect, Figure S1: Detection of overexpression efficiency of the *PDGF-D* gene. (**A**) Positive cells observed using a light microscope (20×). (**B**) Positive cells observed using a fluorescence microscope (20×). (**C**) Relative mRNA expression levels of the *PDGF-D* gene.

## Abbreviations

The following abbreviations are used in this manuscript:

ACTB	β-actin
He	heterozygosity
LPL	lipoproteinlipase
MALDI-TOF-MS	matrix-assisted laser desorption/ionization time-of-flight mass spectrometer
Ne	effective allele number
PDGF-D	platelet-derived growth factor-D
PIC	polymorphic information content
PPARγ	peroxisome proliferator-activated receptor gamma
qRT-PCR	quantitative real-time PCR
SNP	single nucleotide polymorphism
TF	transcriptional factor
