# Supplementary material for: Verification and Analysis of Sheep Tail Type-Associated PDGF-D Gene Polymorphisms
Source: Animals (Basel). 2020 Jan 6;10(1):89. doi: 10.3390/ani10010089 (PMC7022463; doi:10.3390/ani10010089)
Supplement: Supplementary file 1 [file animals-10-00089-s001.zip › Supplementary File(s)/Supplementary Table S1.docx]

**Table S1** **Primers used in this study for PCR.**

| Primer | Primer sequence | Tm (°C) | Length (bp) | Locus |
| --- | --- | --- | --- | --- |
| *PDGF-D*-1F | ATGCGTGGCAAAACCAATAC | 60 | 1006 | 5' UTR -0-500bp |
| *PDGF-D*-1R | GCCTCAGCCCTAGACATCAG |  |  |  |
| *PDGF-D*-2F | GGGAGACCTAGGCTTGATCC | 59 | 919 | 5' UTR-500-1000bp |
| *PDGF-D*-2R | GCGTCACTCCAAGAAAAAGC |  |  |  |
| *PDGF-D*-3F | CTGTATGGTCCATGGGGTCT | 58 | 1029 | Exon1 |
| *PDGF-D*-3R | CTCTCCCACACACACACCAC |  |  |  |
| *PDGF-D*-4F | AGTGAAAGGGAAGTCGCTCA | 58 | 626 | Exon2 |
| *PDGF-D*-4R | TCATTTGTTCTCACCCCAAA |  |  |  |
| *PDGF-D*-5F | AACCGCAAAGCTGCTATTTG | 60 | 604 | Exon3 |
| *PDGF-D*-5R | AGATCCCCCTTCTCTGGAAA |  |  |  |
| *PDGF-D*-6F | AGTAGTCTGGCCTGGGGAAT | 60 | 631 | Exon4 |
| *PDGF-D*-6R | GCAGGGACAAACCATACCAC |  |  |  |
| *PDGF-D*-7F | AGCAGGCGTGTAGCCTGTAT | 60 | 540 | Exon5 |
| *PDGF-D*-7R | TCAAAATTCCCCACTTGAGC |  |  |  |
| *PDGF-D*-8F | TTGAGGCTAAGCAAGGGAGA | 60℃ | 996 | Exon6 |
| *PDGF-D*-8R | GAGGCAAGAGACCGTGATTC |  |  |  |
| *PDGF-D*-9F | GGTGCAAGAAAAATTGAACCA | 57 | 918 | Exon7 |
| *PDGF-D*-9R | GACTGTTGTCAACCCGCATA |  |  |  |
| *PDGF-D*-10F | TCAGACACCTGTGCTCCTCA | 60 | 1024 | Exon8-1 |
| *PDGF-D*-10R | TTGTGATGACATGCTTTTGGA |  |  |  |
| *PDGF-D*-11F | CACCAGCAACCGAACTTACT | 60 | 1203 | Exon8-2 |
| *PDGF-D*-11R | CCATGGACTGTAGCCTCCTC |  |  |  |
| *PDGF-D*-12F | AGCCGAAGCAGAATTTTGAA | 60 | 1167 | Exon8-3 |
| *PDGF-D*-12R | GGCTGCTCATCTGACACTGA |  |  |  |
| *PDGF-D*-13F | ACCTGGGGTCTCTGGTTCTT | 60 | 1696 | Exon8-4 |
| *PDGF-D*-13R | CACCGACTGTGGTGTTCATC |  |  |  |
| *PDGF-D*-14F | GGAATAAAAAGCTCACCGTACAA | 59 | 1103 | 3' UTR-0-500bp |
| *PDGF-D*-14R | CAACAAGGCACACTCCAAAC |  |  |  |
| *PDGF-D*-15F | GTGTCCACAGGCTGTCTCCT | 60 | 910 | 3' UTR-500-1000bp |
| *PDGF-D*-15R | GGACATGTGTGCATTTCAGG |  |  |  |
