# Supplementary material for: Verification and Analysis of Sheep Tail Type-Associated PDGF-D Gene Polymorphisms
Source: Animals (Basel). 2020 Jan 6;10(1):89. doi: 10.3390/ani10010089 (PMC7022463; doi:10.3390/ani10010089)
Supplement: Supplementary file 1 [file animals-10-00089-s001.zip › Supplementary File(s)/Supplementary Table S2.docx]

**Table S2** **Primers used in this study for qRT-PCR.**

| Gene | Primer | Primer sequence | Tm | Length |
| --- | --- | --- | --- | --- |
| *PDGF-D* | *PDGF-F* | *GCGGATGCTCTGGACAAA* | *59* | *218* |
|  | *PDGF-R* | *AGGCAGCGTGGAAAGAAG* |  |  |
| *PPARγ* | *PPAR-F* | *ATAAAGCGTCAGGGTTCCAC* | *60°C* | *115 bp* |
|  | PPAR-R | ATCCGACAGTTAAGATCACACC |  |  |
| *LPL* | *LPL*-F | AGACTCGTTCTCAGATGCCTT | 63°C | 127 bp |
|  | LPL-R | CTCTCAGCCACAGTGCCAT |  |  |
| *β-Actin* | Actin-F | CCAACCGTGAGAAGATGACC | 60°C | 97 bp |
|  | Actin-R | CCCGAGGCGTACAGGGACAG |  |  |
