# Supplementary material for: Verification and Analysis of Sheep Tail Type-Associated PDGF-D Gene Polymorphisms
Source: Animals (Basel). 2020 Jan 6;10(1):89. doi: 10.3390/ani10010089 (PMC7022463; doi:10.3390/ani10010089)
Supplement: Supplementary file 1 [file animals-10-00089-s001.zip › Supplementary File(s)/Supplementary Table S3.docx]

**Table S3** **The breed effect.**

| Locus | Traits | Breed effect |
| --- | --- | --- |
| g.4122606 C>G | Tail length | < 0.001 |
|  | Tail width | < 0.001 |
|  | Tail circumference | < 0.001 |
| g.3852134 C>T | Tail length | < 0.001 |
|  | Tail width | < 0.001 |
|  | Tail circumference | < 0.001 |
